# Editorial: Immunosenescence and multiple sclerosis: Prognostic and therapeutic implications

**DOI:** 10.3389/fneur.2022.996342

**Published:** 2022-08-30

**Authors:** Aurora Zanghì, Carlo Avolio, Hans-Peter Hartung, Emanuele D'Amico

**Affiliations:** ^1^Unitá Operativa Complessa (UOC) Neurology, Sant'Elia Hospital, Caltanissetta, Italy; ^2^Department of Medical and Surgical Sciences, University of Foggia, Foggia, Italy; ^3^Department of Neurosciences, Head of Multiple Sclerosis Center, Policlinico Riuniti Hospital, Foggia, Italy; ^4^Department of Neurology, Medical Faculty, Heinrich-Heine-University Düsseldorf, Düsseldorf, Germany; ^5^Brain and Mind Center, The University of Sydney, Sydney, NSW, Australia; ^6^Department of Neurology, Palacky University Olomouc, Olomouc, Czechia

**Keywords:** multiple sclerosis, disease modifying therapies, age, inflammaging, immunosenescence

Multiple sclerosis (MS) is a multifaceted disorder that mainly affects young adults, and it dramatically impacts their work and social abilities. The so-called late-onset MS (LOMS) is still considered very rare with a prevalence ranging from 4 to 9.4% according to different cohort studies (1, 2). LOMS displays several differences compared to young onset MS in terms of clinical and paraclinical characteristics, and it seems that LOMS more frequently presents a progressive course from the onset with a shorter time to severe motor disability; this could be associated with the higher incidence of comorbidities and polypharmacy (3, 4). As MS is a prototypic autoimmune disease of the central nervous system, we have to take into account how the immune system presents profound changes, both quantitatively and functionally, during an individual's lifetime, and it is important to understand the differences between these patients and younger patients.

Immunosenescence is defined as age-related changes in the immune system, leading to an increase in morbidity and mortality in older adults (5). The most important processes of immunosenescence are associated with a decrease in the number of naive T and B cells, NK cells, and disruption of the pro- and anti-inflammatory balance by changes in the production of cytokines (5).

The approval of a new disease modifying treatment (DMTs) for MS and the aging of population makes it increasingly important that we attempt to explain how the immune system alterations can be additive to, or work in synergy with, the changes induced by DMTs concerning the risk of infections and the disease course (activity and progression).

The aim of this Research Topic has been to provide new insights into the immunosenescence phenomenon in the MS population to identify how aging can influence disease trajectory and response/tolerability to DMTs (6).

The Authors have contributed with 12 valuable works on different aspects of immunosenescence in MS, including two reviews and two mini-reviews.

In particular, Li et al. analyzed and compare different clinical, laboratory, and magnetic resonance imaging characteristics between pediatric and adult patients with first-attack myelin oligodendrocyte glycoprotein antibody disease (MOGAD) to explore predictive factors for severity at disease onset. The authors found the clinical phenotype of MOGAD varies in patients of different ages (Li et al.).

Focusing on T-cell senescence, Tomas-Ojer et al. investigated the involvement of antigen-induced T-cell senescence in controlling CD4+ T-cell-mediated autoimmune responses in MS. Here, patients with high levels of CD4+ T-cell senescence in peripheral blood showed increased frequencies of CSF-infiltrating CD28+ CD27-EM CD4+ T cells with a proinflammatory Th1 functional phenotype. The correlation of these cells with the intrathecal levels of neurofilament light chain, a marker of neurodegeneration, suggests their relevance in disease pathogenesis and the involvement of T-cell senescence in their regulation. Markers of antigen-induced T-senescence, therefore, could promise as a tool to identify pathogenic CD4+ T cells in patients with MS (Tomas-Ojer et al.).

Perdaens and van Pesch focused on consequences of age-related immune changes on MS pathology in terms of interaction with the intrinsic aging process of central nervous system resident cells, and then they discussed the impact of immunosenescence on disease evolution and the safety and efficacy of current DMTs.

Furthermore, Ysrraelit and Correale discussed the role of androgens in the development and function of the innate and adaptive immune response as well as in neuroprotective mechanisms relevant to MS; evidence of epidemiological studies has shown a later age of onset of MS in men, relative to women, which could perhaps correspond to the decline in protective testosterone levels (Ysrraelit and Correale).

Manouchehri et al. discussed the role of immune senescence on different arms of the immune system and how it may explain relative DMT resistance based on the classical dichotomy of DMT effectiveness between relapsing MS and progressive MS, which is informative of distinct pathogeneses of the different MS phenotypes (7).

The use of DMT in the elderly population has been investigated by Ng et al.. They conducted a population-based observational study using linked administrative health data from British Columbia, Canada. Their results showed that any DMT, vs. no DMT, in the <55-year-olds was associated with a 23% lower hazard of hospitalization (adjusted hazard ratio, aHR 0.77; 95% CI 0.72–0.82), but not in the ≥55-year-olds (aHR 0.95; 95% CI 0.87–1.04) (Ng et al.).

Buscarinu et al. discussed how the aging process influences the onset, the clinical course, and the therapeutic approach in LOMS.

Giovannoni et al. performed an Integrated Lymphopenia Analysis in Younger and Older patients treated with Cladribine (Clad) at a dosage of 3.5 mg/kg, focusing on the possible effect of Clad on lymphocyte levels by age. Overall, lymphocyte recovery began soon after nadir following Clad treatment and median levels reached normal range by end of the treatment year in both age groups. The rate of certain infections was numerically higher in older vs. younger patients (Giovannoni et al.). In conclusion, Clad had a similar effect on ALC and lymphocyte subsets in both younger and older patient groups (Giovannoni et al.).

Vollmer et al. in a real-world cohort of relapsing MS patients, revealed high-efficacy DMTs had less benefit with aging but were associated with increased risks. These results help overcome some limitations of trials where older patients were excluded. To better balance benefits/risks, authors have proposed a DMT de-escalation approach for aging MS patients (Vollmer et al.).

Real-world data and post-marketing surveillance are certainly of interest since many patients who started DMT over the last decades are currently older than the age limits usually used in clinical trials. Wandall-Holm et al. proposed the results from a Danish MS cohort and revealed patients with MS are at a higher risk of losing all income from earnings and at a much higher risk of receiving disability pension compared with healthy controls.

This special issue warrants further study specifically designed to quantify risk and to disclose better strategies to minimize such a risk.

## Author contributions

AZ: conceptualization, methodology, project administration, and writing—original draft preparation. CA and H-PH: conceptualization and writing—original draft preparation. ED'A: conceptualization, methodology, writing—original draft preparation, writing—review and editing, supervision, and final validation. All authors contributed to the article and approved the submitted version.

## Conflict of interest

The authors declare that the research was conducted in the absence of any commercial or financial relationships that could be construed as a potential conflict of interest.

## Publisher's note

All claims expressed in this article are solely those of the authors and do not necessarily represent those of their affiliated organizations, or those of the publisher, the editors and the reviewers. Any product that may be evaluated in this article, or claim that may be made by its manufacturer, is not guaranteed or endorsed by the publisher.
